# Effect of two-photon absorption on trapping of plasmonic nanoparticles

**DOI:** 10.1038/s41598-024-63235-0

**Published:** 2024-06-01

**Authors:** S. Mirzaei-Ghormish, K. Qaderi, D. Smalley

**Affiliations:** Department of Electrical and Computer Engineering, Brigham Young University, Provo, UT USA

**Keywords:** Optics and photonics, Nonlinear optics

## Abstract

In this paper, we introduce a theoretical framework for optical trapping that integrates nonlinear polarization within the dipole approximation. This theory represents the most comprehensive analytic model to date capable of resolving the discrepancies between the observed and simulated trapping of plasmonic nanoparticles. Our theory elucidates how two-photon absorption can account for the stable trapping of gold nanoparticles, including their longitudinal stability, especially near their plasmon resonance. Furthermore, the experimentally observed split potential wells in the transverse plane, which are attributed to two-photon absorption, are in close agreement with our model’s predictions. Finally, this study provides new insights into the mechanism of optical trapping under conditions of intense light–matter interactions.

## Introduction

Plasmonic nanoparticles exhibit unique properties, such as localized surface plasmon resonance, rendering them highly useful in color manipulation^1^, enhanced Raman spectroscopy^2^, cancer treatment^3^, and catalysis^4^. Optical levitation further enhances their utility by enabling their precise control in three-dimensional space and by isolating them from environmental interference. While plasmonic nanoparticles hold immense promise, their optical trapping, both experimentally and theoretically, presents significant challenges due to the intricate interaction between their unique optical characteristics and the trapping laser.

One significant challenge lies in the discrepancies between current theoretical predictions and experimental observations. For instance, contrary to the predictions of linear theory, experimental studies—often conducted in the infrared spectrum—have demonstrated the stable trapping of plasmonic nanoparticles larger than 80 nm under illumination by a single laser beam^5–7^. This outcome is particularly unexpected in the longitudinal direction, where linear theory predicts the absence of a potential well. In an attempt to reconcile these experimental observations, one hypothesis suggests the presence of steam nanobubbles around the trapped nanosphere^8^. This notion requires further examination, as recent studies have shown that gold nanoparticles can reach their melting temperature without any associated nanobubble formation^9^. The observed stability has also been attributed to non-spherical particle shapes, which enhance the longitudinal stiffness of the trap^10^. However, it is important to note that this explanation holds true only for certain non-spherical shapes and specific orientations relative to the incoming laser beam.

The complexity intensifies near plasmon resonance wavelengths, where conventional trapping mechanisms may no longer hold^11^. It has been argued that surface creeping waves can explain the stable trapping of plasmonic particles near the plasmon resonances^12^. While the concept of exciting surface creeping waves effectively explains transverse stability, it does not account for longitudinal stability on the optical axis of the laser beam. It could be argued that in proximity to the particle plasmon resonance, the conventional description of optical trapping no longer applies and that a new model is needed.

**Figure 1:**
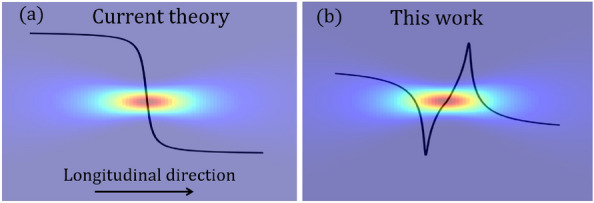
The comparison between (**a**) linear (current theory) and (**b**) nonlinear (this work) models for longitudinal potential traps. The linear model predicts an unstable potential trap, while the nonlinear model predicts a stable potential trap where the stable point is located on the left side of the focal point.

To address these discrepancies, we propose the first analytic model that fully incorporates all necessary nonlinear optical effects, especially two-photon absorption (TPA), which plays a pivotal role in resolving the inconsistencies between theory and experiment. Our theoretical framework, based on considering nonlinear polarization within the dipole approximation, demonstrates that TPA accounts for the stable trapping of gold nanoparticles near plasmon resonance. In the longitudinal direction, an asymmetric potential arises, leading to stable points positioned ahead of the focal point of a tightly focused Gaussian beam, see Fig. 1.

Our framework also extends understanding of trapping in the transverse plane. Several prior theories, considering electronic Kerr nonlinearity and utilizing various approximations (discarding scattering force, self-induced back-action, and TPA), have attempted to explain the experimental observation of split traps in the transverse plane^13–16^, but as this simplified analysis is made more complete with the reintroduction of scattering force and back-action, split potentials begin to vanish^14^, only to reappear again an even more complete numerical force calculation including complex third-order susceptibility^17^. This is consistent with strong experimental evidence suggesting that split traps occur due to TPA^13^ The nonlinear numerical analysis does not; however, isolate TPA from Kerr nonlinearity as the source of split traps nor does it provide insight into the mechanism of the nonlinear trapping system available in an analytic approach. Our model, grounded in a power expansion of polarizability not only aligns with experimental results but also offers a useful physical interpretation of nonlinear optical trapping systems. It highlights the significant impact of nonlinear effects, especially two-photon absorption (TPA), on trapping plasmonic nanoparticles. This study bridges the gap between theory and experiment, providing insights into the mechanisms at play. Furthermore, by drawing an analogy with a nonlinear harmonic oscillator, we deepen the understanding of the system’s behavior.

## Theory

Gold nanoparticles exhibit strong third-order nonlinearity at plasmon resonances^18^. To explore the effects of this nonlinearity on trapping, our analysis assumes a linearly polarized continuous laser beam ( wavelength $\lambda =532$ nm, numerical aperture NA = 1.4 ) that propagates in the *z*-direction to illuminate gold nanoparticles (radius $R=40$ nm) in air. Under the influence of a high-intensity continuous-wave laser or pulsed laser, the induced nonlinear polarizability becomes significant and must be taken into account in the computation of optical forces. The relative permittivity of the particle, including the third-order nonlinearity, is written as, $\epsilon _{p}=1+\chi _{1}+3\chi _{3}E^{2}$, where $\chi _{1}=\chi ^{\prime }_{1}+i\chi ^{\prime \prime }_{1}$ is the complex linear susceptibility, $\chi _{3}=\chi ^{\prime }_{3}+i\chi ^{\prime \prime }_{3}$ is the complex nonlinear susceptibility, in which the real part corresponds to electronic Kerr nonlinearity, the imaginary part relates to TPA, and *E* is the amplitude of the electric field. The third-order nonlinearity of gold originates from interband transitions, intraband transitions, and hot electrons^19^. Both intraband and interband transitions have an instantaneous response, while hot electrons exhibit a response time on the order of hundreds of femtoseconds.

The details of theoretical calculations are presented in the Supplemental Information. Here we present the final equations for real ($\alpha ^{\prime }$) and imaginary ($\alpha ^{\prime \prime }$) parts of effective susceptibilities in weakly absorbing conditions.

In low absorption limit ($\chi ^{\prime \prime } \ll \chi ^\prime$, $\chi _3^{\prime \prime }\ll \chi _3^\prime$), we get

1 $$\begin{aligned} \alpha ^{\prime }&= 4\pi \epsilon _oR^3\frac{\epsilon ^{\prime }_{p}-1}{\epsilon ^{\prime }_{p}+2} + 36\pi \epsilon _oR^3E^2\chi _3^{\prime } \frac{\epsilon ^{\prime }_{p}-1}{(\epsilon ^{\prime }_{p}+2)^2} \end{aligned}$$

2 $$\begin{aligned} \alpha ^{\prime \prime }&= \alpha _1^{\prime \prime }+12\pi \epsilon _0R^3 \frac{\chi ^{\prime \prime }+3E^2\chi _3^{\prime \prime }}{(\epsilon ^{\prime }_{p}+2)^2} \end{aligned}$$

and

3 $$\begin{aligned} \alpha _1^{\prime \prime } = \frac{8}{3}\pi \epsilon _ok^3R^6 \left( \frac{\epsilon ^{\prime }_{p}-1}{\epsilon ^{\prime }_{p}+2}\right) ^2 + 48\pi \epsilon _ok^3R^6E^2\chi _3^{\prime } \frac{\epsilon ^{\prime }_{p}-1}{(\epsilon ^{\prime }_{p}+2)^3} \end{aligned}$$

where, $\epsilon _{p}=\epsilon _{p}^{\prime }+i \epsilon _{p}^{\prime \prime }$ is the relative permittivity of the particle. In this condition, the Kerr nonlinearity contributes to both gradient and scattering forces. Conversely, TPA solely contributes to the scattering force. The ratio of the Kerr effect to TPA components in Eq. (1) is proportional to $(kR)^3\left( \frac{\chi ^{\prime }_3}{\chi ^{\prime \prime }_3}\right)$, where $(kR)^3 \ll 1$ and $\left( \frac{\chi ^{\prime }_3}{\chi ^{\prime \prime }_3}\right) \gg 1$. As a result, in certain circumstances, the Kerr effect dominates, whereas in others, TPA primarily contributes to the scattering force. Depending on the values of $\chi ^{\prime }_{3}$ and $\chi ^{\prime \prime }_{3}$, as well as the magnitude of the input power, the Kerr effect and TPA effects can either increase or decrease the gradient and scattering forces. Hence, the new potential traps other than the harmonic quadratic potential can be achieved.

From Eqs. (1)–(3), one can observe that both the real and imaginary parts of susceptibility are decomposed into linear and nonlinear components. This implies that the total system can be considered a combination of linear and nonlinear oscillators. In the next section, we discuss this in more detail.

**Figure 2:**
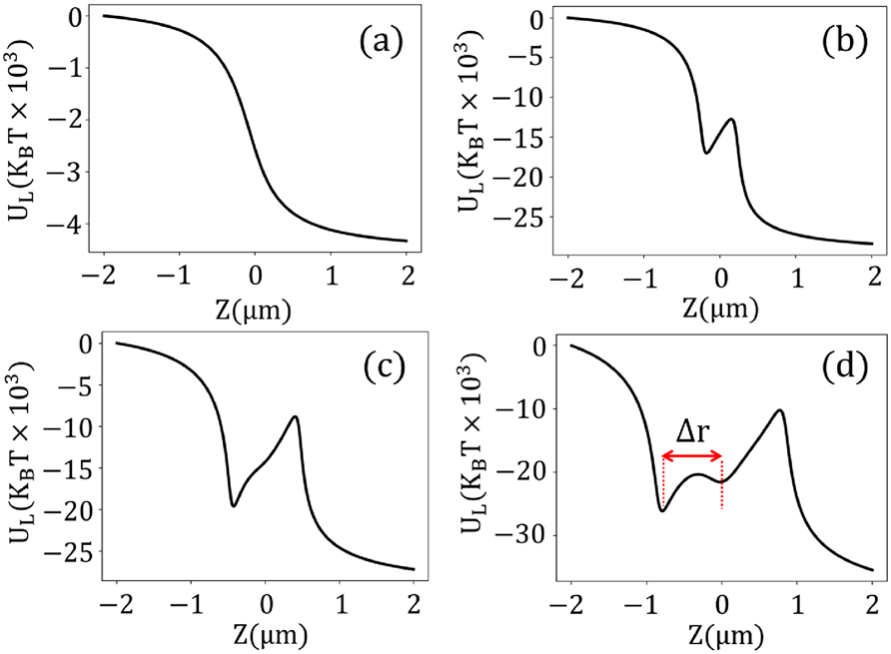
Longitudinal potential for (**a**) $P_{ave}= 100$ mW, (**b**) $P_{ave}= 500$ mW, (**c**) $P_{ave}= 1000$ mW, and (**d**) $P_{ave}= 2500$ mW, taking into account both Kerr nonlinearity and two-photon absorption.

We first investigate the longitudinal stability on the optical axis of the laser beam. For the following analysis, we consider both Kerr and TPA together before finally considering Kerr effects alone (noting their insufficiency). In this way, we can isolate the unique contributions of TPA. Figure 2 presents the longitudinal potential ($\text {U}_{\textrm{L}}$) at different powers, taking into account a complex value for the third-order susceptibility of gold, i.e. $\chi _{3}=(3.5-2.5j)\times 10^{-16}\, (m^2/V^2)$^18^. Additionally, the corresponding forces ($\text {F}_{\textrm{L}}$) are detailed in the Supplemental Information. At low powers ($P_{ave}\le 300\, \text {mW}$), the effect of optical nonlinearities is not significant, and the trapping system behavior approaches that of the linear regime. In a linear regime, no potential depth is created, and gold nanoparticles exhibit instability in the longitudinal direction (Fig. 2a). As the power increases, the nonlinear effects become more obvious, leading to longitudinal stability. At higher powers ($P_{\textrm{ave}} > 300\, \text {mW}$), an asymmetric potential well emerges, with the stable point situated to the left of the focal point, as depicted in Fig. 2b–d. The shape of these potential wells differs from conventional potential traps, which exhibit harmonic quadratic behavior with stable points centered at the focal point. Additionally, an extra depth tends to appear at the focal point at extremely high powers of $P_{\textrm{ave}} = 2500 \,\text {mW}$.

The position and width of the longitudinal trap potential vary with changing the input power. As the power increases, both the width and depth of the trap increase, and the position of the stable point progressively moves farther away from the focal point (see Fig. 4a). One could say that the nonlinear force acts as a pulling force, drawing the trapped particles toward the laser source.

Note that the longitudinal stability of plasmonic particles at wavelengths further from the plasmon resonance can be justified without considering the effect of optical nonlinearities. In these regimes (infrared), plasmonic particles behave like weakly absorbing particles, and the attractive gradient forces can dominate the repulsive scattering forces under tightly focused laser illumination.

**Figure 3:**
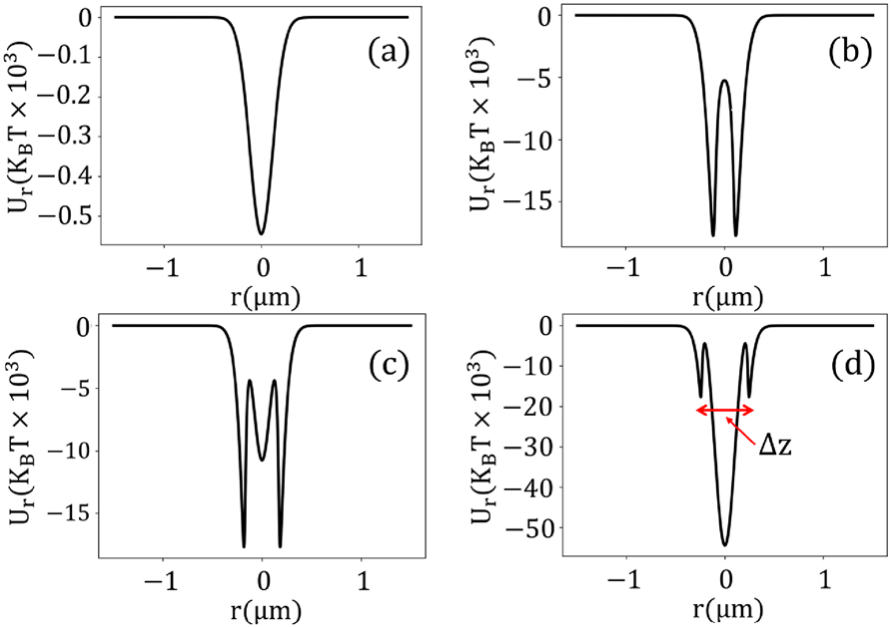
Transverse potential energy for (**a**) $P_{ave}= 100$ mW, (**b**) $P_{ave}= 500$ mW, (**c**) $P_{ave}= 1000$ mW, and (**d**) $P_{ave}= 2500$ mW, accounting for two-photon absorption in conjunction with Kerr nonlinearity.

Figure 3 shows the transverse potential energies for different powers when considering both electronic Kerr nonlinearity and TPA. Moreover, the corresponding forces are presented in the Supplemental Information. As shown, the gold nanoparticles are stably trapped in the transverse plane. At lower powers ($P_{ave}\le 300$ mW), the potential energy resembles a quadratic shape, similar to the linear potential energy. As the power increases ($P_{ave}> 300$ mW) due to nonlinear effects, bistable traps occur with two wells symmetrically positioned on opposing sides of the focal point (Fig. 3b). The depths of these wells significantly exceed the kinetic energy of Brownian motion, ensuring stable trapping. An increase in power increases the potential barrier between the split traps, further impeding particle transitions between the traps. At ultra-high powers ($P_{ave}>1000$ mW), a tri-stable potential trap is created with three wells: one central and two symmetrically positioned on either side (Fig. 3c, d). We established that the total system can be viewed as a composite of linear and nonlinear oscillators. While the linear potential exhibits a single well at the origin, the nonlinear potential introduces two symmetrical off-center wells. As the power increases, the potential barrier separating these off-center wells surpasses the depth of the linear potential. In extremely high powers, a reverse effect occurs so that the linear potential depth overcomes the potential barrier, creating a third central well. Compared to the central potential well (linear potential), the off-center wells (nonlinear potentials) have a narrower width, indicating that the stiffness of the nonlinear oscillator is greater than that of the linear one. Interestingly, the depth of the off-center wells remains constant regardless of input power (Fig. 3b, c). This shows that the stability of off-center trap points does not depend on power, instead it depends only on linear and nonlinear susceptibilities. This contrasts with the central well which depends on both power, linear, and nonlinear susceptibilities. In the next section, We discuss these results mathematically.

**Figure 4:**
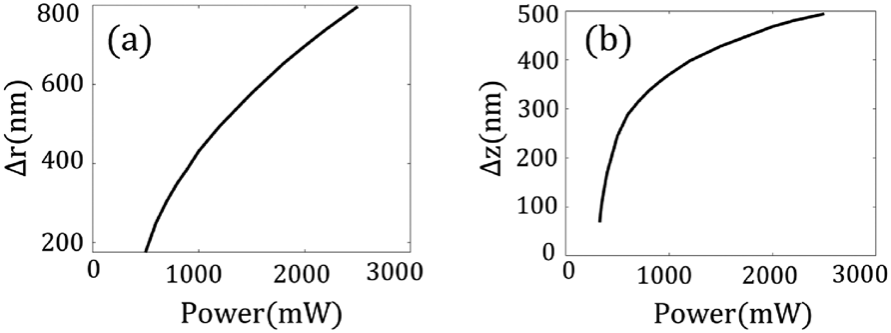
(**a**) The positioning of trap points as a function of power in the longitudinal direction. (**b**) The spacing between two split traps as a function of power in the transverse plane.

The spacing between the two off-center split traps expands with increasing power as depicted in Fig. 4b. Later we show that it follows a logarithmic form. Moreover, the spacing can surpass the diffraction limit. For instance, at an input power of $P_{ave}= 330$ mW, this distance is 74 nm.

**Figure 5:**
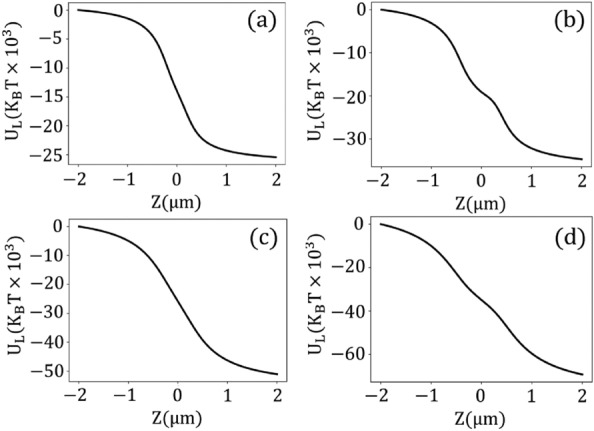
Longitudinal potential energy for (**a**) $P_{ave}= 500$ mW, and (**b**) $P_{ave}= 1000$ mW in air. Longitudinal potential energy for (**c**) $P_{ave}= 500$ mW, and (**d**) $P_{ave}= 1000$ mW in water. In both cases, only the effect of Kerr nonlinearity is considered.

**Figure 6:**
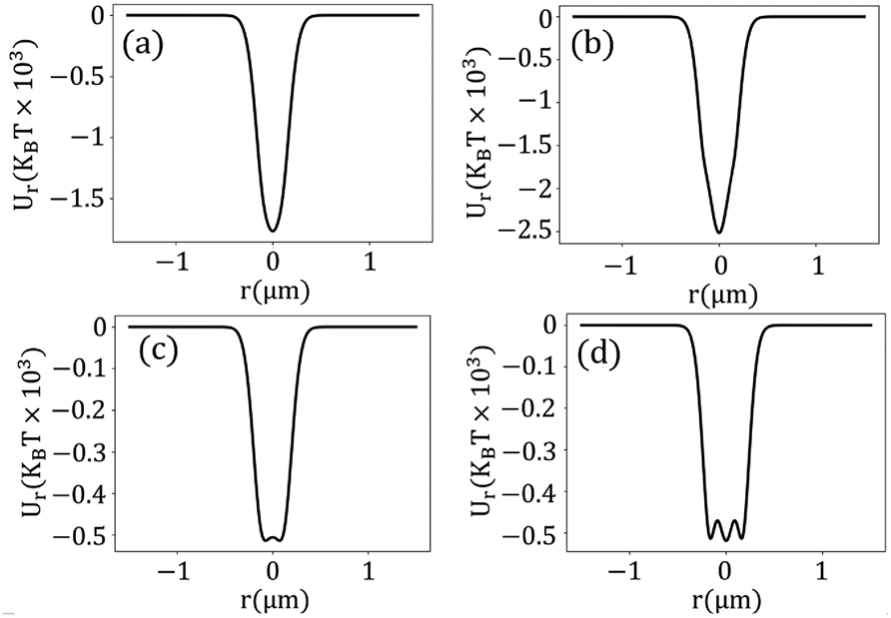
Transverse potential energy for (**a**) $P_{ave}= 500$ mW, and (**b**) $P_{ave}= 1000$ mW in air. Transverse potential energy for (**c**) $P_{ave}= 500$ mW, and (**d**) $P_{ave}= 1000$ mW in water. In both cases, only the effect of Kerr nonlinearity is considered.

In what follows, we only investigate the influence of electronic Kerr nonlinearity on the optical trapping of gold nanoparticles. Figs. 5 and 6, respectively, show the longitudinal and transverse potential energies when considering $\chi _3^{\prime \prime }=0$. The insets a and b represent the results for a particle located in air, while insets c and d represent the results for a particle located in water. The corresponding forces are again shown in the Supplemental Information. As shown, the gold nanoparticles are unstable in both air and water in the longitudinal direction when considering only the Kerr effect. While the Kerr nonlinearity introduces a slight perturbation to the longitudinal potentials at high powers, the trap retains its instability.

In the transverse plane, split potential traps do not manifest for gold nanoparticles located in the air, even at high powers. This indicates that the sole influence of electronic Kerr nonlinearity is not significant to establish optical trapping. This insufficiency extends to gold nanoparticles immersed in water, where the potential energy splits into two shallow wells symmetrically positioned on either side of the focal point at $P_{ave}\ge 500$ mW. At higher powers $P_{ave}\ge 1000$ mW, the potential energy splits into three shallow wells: a central one at the focal point and two equidistant ones around the origin. These localized potential wells are superficial and are not deep enough to overcome Brownian motion, preventing particles from remaining confined. Once trapped in one well, Brownian motion easily moves particles to transition between wells.

The theoretical calculations reveal that the Kerr nonlinearity process alone has little contribution to forming stable split traps observed experimentally. Even though it introduces shallow split traps in the transverse plane, these traps lack stability. Moreover, it does not contribute to the longitudinal stability. Alone Kerr nonlinearity contradicts experimental observations wherein particles are consistently trapped within split traps on the transverse plane or stably positioned longitudinally^13^; however, when combined with TPA the theory becomes consistent with the experiment. Thus by eliminating Kerr nonlinearity as a contributor, we can with greater confidence attribute the origin of deep split traps, as well as longitudinal stability, to TPA.

In response to prior theories^14–17^ that attribute split traps to electronic Kerr nonlinearity instead of the two-photon absorption observed experimentally, we respectfully suggest that this might be a misinterpretation. This potential error could stem from oversimplifications in the force calculations, which overlook scattering forces and self-induced back-action. It is also possible that the effect of these omissions may be further compounded by the arbitrary assignment of values for Kerr nonlinearity for the chosen target wavelength.

## Physical interpretation

In this section, we adopt a different approach to model the transverse trapping potential and demonstrate that this new interpretation aligns entirely with the results presented in the previous section. We first revise the previous theories of nonlinear optical trapping presented in^14–17^, by incorporating TPA into the calculations. Then, we offer a physical interpretation for these nonlinear trapping systems.

As demonstrated in the previous section, at some powers, the transverse potential traps exhibit behavior similar to the bi-stable potential of a nonlinear oscillator. At ultra-high powers, the system behaves like a tri-stable potential. As previously demonstrated, the total trapping potential is a combination of linear and nonlinear potential traps. Accordingly, the bi-stable and tri-stable potential energies can be presented by the equations $U_{bi}=-\frac{1}{2}k_{1}x^2+\frac{1}{4}k_{2}x^4$, and $U_{tri}=\frac{1}{2}k_{1}x^2-\frac{1}{4}k_{2}x^4+\frac{1}{6}k_{3}x^6$, respectively. Here, $k_{1}$ is linear stiffness and $k_{2}$ and $k_{3}$ denote nonlinear stiffnesses. To better understand the mechanism of nonlinear trapping in the transverse plane, we make some simplifications. Specifically, we neglect the scattering force and self-induced back action. These assumptions are valid for weakly absorbing particles trapped in water which have a reduced scattering cross-section in the transverse plane.

The details of calculations are presented in Supplemental Information. After simplifications, the total transverse potential can be expressed as a combination of linear (first part) and nonlinear (second part) counterparts.

4 $$\begin{aligned} U = -E_0^2 e^{-2\rho ^2} + \frac{3(3+\chi ^{\prime })}{(3+\chi ^{\prime })^2 +(\chi ^{\prime \prime })^2}E_0^2 e^{-2\rho ^2} \end{aligned}$$

where $\chi ^{\prime }=\chi ^{\prime }_{1}+\chi ^{\prime }_{3}$ and $\chi ^{\prime \prime }=\chi ^{\prime \prime }_{1}+\chi ^{\prime \prime }_{3}$. According to Eq. (4), the envelope of nonlinear potential is the linear counterpart. The linear potential has a depth at its central point. By adding the nonlinear potential, the total potential can exhibit multiple depths depending on the values of linear and nonlinear susceptibilities.

The magnitude of total potential at the focal point, i.e. $U(\rho =0)=U_{L}(\rho =0)+U_{NL}(\rho =0)$ reads

5 $$\begin{aligned} U(0) = -E_0^2+ \frac{3(3+\chi _1^{\prime } + 3\chi _3^{\prime }E_0^2)}{(3+\chi _1^{\prime } + 3\chi _3^{\prime }E_0^2)^2 +(\chi _1^{\prime \prime } + 3\chi _3^{\prime \prime }E_0^2)^2}E_0^2 \end{aligned}$$

Here, the first term represents the depth of the linear potential, and the second term corresponds to the amplitude of the nonlinear potential barrier, with $E_0$ being the maximum amplitude of the electric field. As the power increases, the depth of the linear potential deepens, while the potential barrier of the nonlinear potential initially rises (in the saturable absorption regime) before converging to a fixed value (in the reverse saturable absorption regime). In the saturable absorption regime, the magnitude of the second term dominates the first one, leading to positive amplitudes as confirmed in Fig. 3b. Conversely, in the reverse saturable absorption regime, the magnitude of the nonlinear term at the focal point is independent of power, i.e,

6 $$\begin{aligned} \lim _{E_0\rightarrow \infty } U_{NL}(\rho =0)= \frac{\chi _3^{\prime }}{(\chi _3^{\prime })^2+(\chi _3^{\prime \prime })^2} \end{aligned}$$

In contrast, the depth of the linear potential continues to deepen. Thus, the magnitude of the linear component prevails over the nonlinear one, resulting in the emergence of a centered well, as depicted in Figs. 2d, and 3c, d. On the other hand, the off-center trap points happen at

7 $$\begin{aligned} \rho _{\pm } = \pm \sqrt{Ln\left( \frac{3(\chi _3^{\prime } -\chi _3^{\prime \prime })E_0^2}{\chi _1^{\prime \prime } -\chi _1^{\prime }-3}\right) ^{\frac{1}{2}}} \end{aligned}$$

where $\rho _{\pm }$, respectively, are right and left off-center points. From Eq. (7) multi-split traps do not appear for arbitrary values of linear and nonlinear susceptibilities. The condition for forming a split trap is that the argument of the logarithmic function must be greater than one; i.e. the relation between power, linear and nonlinear susceptibilities should be $\frac{3(\chi _3^{\prime }-\chi _3^{\prime \prime }) E_0^2}{\chi _1^{\prime \prime }-\chi _1^{\prime }-3}>1$, to see split traps.

According to Eq. (7), the distance between off-center points is proportional to $\sqrt{Ln({E_0})}$ a behavior illustrated in Fig. 4b through a more exact approach. Moreover, the depth of potential wells at off-centered wells denoted $U(\rho _{\pm })$ is given by

8 $$\begin{aligned} U(\rho _{\pm }) = \frac{1}{2}\frac{\chi _1^{\prime \prime } -(\chi _1^{\prime }+3)}{\chi _1^{\prime \prime }\chi _3^{\prime } -\chi _3^{\prime \prime }(3+\chi _1^{\prime })} \end{aligned}$$

As shown, the depth of the off-center trap points is independent of the input power. This result confirms the behaviors in Figs. 3 and 6. Moreover, in most materials the imaginary part of permittivity is much smaller than its real part, therefore, the first terms of the nominator and denominator are much smaller than the second terms, which indicates that the depth of the off-center traps is primarily determined by TPA. This result also agrees with the findings in Figs. 3 and 6.

By comparing $U_{bi}$ and *U*, the relation between linear and nonlinear stiffnesses ($k_1$ and $k_2$) can be calculated as follows:

9 $$\begin{aligned} \frac{k_1}{k_2} = \rho _{+}=\ln \left( \frac{3(\chi _3^{\prime } -\chi _3^{\prime \prime })E_0^2}{\chi _1^{\prime \prime } -\chi _1^{\prime }-3}\right) ^{\frac{1}{2}} \end{aligned}$$

This equation shows that the ratio between the two stiffnesses is not constant; instead, it varies depending on the power, as well as linear and nonlinear susceptibilities. Furthermore, the value of $\rho _{+}$ (the right off-center point) is on the order of micrometers, indicating that $k_2$ is significantly larger than $k_1$, and as demonstrated earlier, the potential width of off-center traps is correspondingly tighter than that of the central trap. Similar analytical methods can also be applied to a tri-stable potential.

The reconfigurability of nonlinear potentials opens up new opportunities in levitated optomechanics. These split traps offer additional control over trapped particles and can be utilized for multi-particle trapping, which is crucial for expanding the capabilities of optical trap displays^20^. Furthermore, these reconfigurable split traps can be valuable in quantum information, enabling the realization of trapped-ion qubits over long distances^21^.

## Conclusion

This paper presents a new theoretical approach to the nonlinear optical trapping of nanoparticles, emphasizing the pivotal roles of Kerr nonlinearity and two-photon absorption. The research highlights TPA’s key role in longitudinal trapping stability and reveals multiple split traps in diverse absorption settings. It suggests that the third-order nonlinearity of gold nanoparticles at plasmon resonances can explain the stable longitudinal trapping observed experimentally. By comparing the nonlinear trap system to a nonlinear harmonic oscillator, the paper deepens our understanding of dipole-regime trapping generally. Ultimately, this study fills existing gaps and advances the field of optical trapping, enriching insights into light-matter interactions.

### Supplementary Information

Supplementary Information.

## Data Availability

All relevant data generated or analyzed during this study are included in this published article [and its supplementary information files]. Any other data used and/or analyzed during the current study is available from the corresponding author on reasonable request.
