# Supplementary material for: Effect of two-photon absorption on trapping of plasmonic nanoparticles
Source: Sci Rep. 2024 Jun 1;14:12607. doi: 10.1038/s41598-024-63235-0 (PMC11144221; doi:10.1038/s41598-024-63235-0)
Supplement: Supplementary file 1 — Supplementary Information. [file 41598_2024_63235_MOESM1_ESM.pdf]

Nonlinear Optical Trapping of Gold Nanoparticles

S. Mirzaei-Ghormish and D. Smalley

Department of Electrical and Computer Engineering,

Brigham Young University, Provo, UT

(Dated: May 28, 2024)

I. THEORETICAL CALCULATIONS

In this section, we develop the optical force calculations based on nonlinear polarization. Using the dipole approximation, the optical forces for a monotonic wave can be expressed as [1]:

$$\vec{F}(\vec{r}) = \frac{\alpha'}{2}\vec{\nabla}\langle|\vec{E}(\vec{r},t)|^2\rangle + \alpha''\omega\langle\vec{E}(\vec{r},t)\times\vec{B}(\vec{r},t)\rangle \tag{S1}$$

Where $\alpha = \alpha' + i\alpha'' = \alpha_0/(1 - i\frac{\alpha_0 k^3}{6\pi\epsilon_0\epsilon_m})$ denotes the effective polarizability of the particle. Meanwhile, $\alpha_0 = 4\pi\epsilon_0 R^3\frac{\epsilon_p-\epsilon_m}{\epsilon_p+2\epsilon_m}$ stands for the quasi-static polarizability, with $\epsilon_p = \epsilon_p' + i\epsilon_p''$ and ϵ_m being the relative permittivities of the particle and medium, respectively. The first term in equation S1 corresponds to the gradient force, whereas the second term signifies the scattering force. When considering the nonlinear susceptibility, the quasi-static polarizability can be written as follows:

$$\alpha_0 = 4\pi\epsilon_o R^3\frac{\epsilon_p + 3\chi_3 E^2 - \epsilon_m}{\epsilon_p + 3\chi_3 E^2 + 2\epsilon_m} \tag{S2}$$

where ϵ_l is linear permittivity of the particle . For simplicity in the calculations, we consider the medium to be air, thus:

$$\alpha_0 = 4\pi\epsilon_o R^3\frac{\epsilon_p + 3\chi_3 E^2 - 1}{\epsilon_p + 3\chi_3 E^2 + 2} = 4\pi\epsilon_o R^3\frac{(\epsilon_p - 1)(1 + \frac{3\chi_3 E^2}{\epsilon_p - 1})}{(\epsilon_p + 2)(1 + \frac{3\chi_3 E^2}{\epsilon_p + 2})} \tag{S3}$$

In general, the nonlinear parts are smaller than their linear counterparts, i.e. in the complex plane $|\frac{3\chi_3 E^2}{\epsilon_l + 2}| < 1$. Thus, we can expand the power series of the denominator as follows:

$$\alpha_0 = 4\pi\epsilon_o R^3\frac{(\epsilon_p - 1)}{(\epsilon_p + 2)}(1 + \frac{3\chi_3 E^2}{\epsilon_p - 1})(1 - \frac{3\chi_3 E^2}{\epsilon_p + 2}) = 4\pi\epsilon_o R^3\frac{(\epsilon_p - 1)}{(\epsilon_p + 2)}(1 + \frac{3\chi_3 E^2}{\epsilon_p - 1} - \frac{3\chi_3 E^2}{\epsilon_p + 2}) \tag{S4}$$

Moreover, E^4 terms are much smaller than the others. After some simplification, we get:

$$\alpha_0 = 4\pi\epsilon_o R^3\frac{(\epsilon_p - 1)}{(\epsilon_p + 2)} + 12\pi\epsilon_o R^3\chi_3 E^2\frac{3}{(\epsilon_p + 2)^2} \tag{S5}$$

The first and second terms, respectively, are the linear and nonlinear parts of quasi-static polarizability ($\alpha_0 = \alpha_0^{(L)} + \alpha_0^{(NL)}$). The linear static polarizability in terms of susceptibilities is written as

$$\alpha_0^{(L)} = 4\pi\epsilon_o R^3\frac{\chi' + i\chi''}{\chi' + i\chi'' + 3} = 4\pi\epsilon_o R^3\frac{(\chi' + i\chi'')(3 + \chi' - i\chi'')}{(3 + \chi')^2 + (\chi'')^2} \tag{S6}$$

By decomposing into real $\alpha_{0,r}^{(L)}$ and imaginary $\alpha_{0,I}^{(L)}$ components, it can be written as

$$\alpha_{0,r}^{(L)} = 4\pi\epsilon_o R^3 \frac{\chi'(3+\chi') + (\chi'')^2}{(3+\chi')^2 + (\chi'')^2} \tag{S7}$$

$$\alpha_{0,I}^{(L)} = 12\pi\epsilon_o R^3 \frac{\chi''}{(3+\chi')^2 + (\chi'')^2} \tag{S8}$$

On the other hand, the nonlinear static polarizability is

$$\alpha_0^{(NL)} = 12\pi\epsilon_o R^3 E^2 \frac{3\chi_3}{(\epsilon_p+2)^2} = 36\pi\epsilon_o R^3 E^2 \frac{\chi_3}{(\chi+3)^2} \tag{S9}$$

where its real $\alpha_{0,r}^{(NL)}$ and imaginary $\alpha_{0,I}^{(NL)}$ components are calculated as follows:

$$\alpha_{0,r}^{(NL)} = \frac{36\pi\epsilon_o R^3 E^2}{[(3+\chi')^2 + (\chi'')^2]^2} [(3+\chi')^2\chi_3' + 2(3+\chi')\chi''\chi_3'' - \chi_3'(\chi'')^2] \tag{S10}$$

$$\alpha_{0,I}^{(NL)} = \frac{36\pi\epsilon_o R^3 E^2}{[(3+\chi')^2 + (\chi'')^2]^2} [(3+\chi')^2\chi_3'' - 2(3+\chi')\chi''\chi_3' - \chi_3''(\chi'')^2] \tag{S11}$$

By substituting the static polarizability into effective polarizability $\alpha_0/(1 - i\frac{\alpha_0 k^3}{6\pi\epsilon_0})$ we get:

$$\alpha = \frac{\alpha_0^{(L)} + \alpha_0^{(NL)}}{1 - i\frac{k^3}{6\pi\epsilon_0}(\alpha_0^{(L)} + \alpha_0^{(NL)})} = \frac{(\alpha_{0,r}^{(L)} + \alpha_{0,r}^{(NL)}) + i(\alpha_{0,I}^{(L)} + \alpha_{0,I}^{(NL)})}{1 - i\frac{k^3}{6\pi\epsilon_o}\left[(\alpha_{0,r}^{(L)} + \alpha_{0,r}^{(NL)}) + i(\alpha_{0,I}^{(L)} + \alpha_{0,I}^{(NL)})\right]} \tag{S12}$$

and by simplification

$$\alpha = \frac{\left[(\alpha_{0,r}^{(L)} + \alpha_{0,r}^{(NL)}) + i(\alpha_{0,I}^{(L)} + \alpha_{0,I}^{(NL)})\right]\left[1 + \frac{k^3}{6\pi\epsilon_o}(\alpha_{0,I}^{(L)} + \alpha_{0,I}^{(NL)}) + i\frac{k^3}{6\pi\epsilon_o}(\alpha_{0,r}^{(L)} + \alpha_{0,r}^{(NL)})\right]}{\left[1 + \frac{k^3}{6\pi\epsilon_o}(\alpha_{0,I}^{(L)} + \alpha_{0,I}^{(NL)})\right]^2 + \left[\frac{k^3}{6\pi\epsilon_o}(\alpha_{0,r}^{(L)} + \alpha_{0,r}^{(NL)})\right]^2} \tag{S13}$$

The terms associated with $(\alpha_{0,r}^{(NL)})^2$ and $(\alpha_{0,I}^{(NL)})^2$ are much smaller than other parts; therefore, they can be taken out. After simplifications, the real and imaginary parts of effective polarizabilities read

$$\alpha' = \frac{\alpha_{0,r}^{(L)} + \alpha_{0,r}^{(NL)}}{\left[1 + \frac{k^3}{6\pi\epsilon_0}(\alpha_{0,I}^{(L)} + \alpha_{0,I}^{(NL)})\right]^2 + \left[\frac{k^3}{6\pi\epsilon_0}(\alpha_{0,r}^{(L)} + \alpha_{0,r}^{(NL)})\right]^2} \tag{S14}$$

$$\alpha'' = \frac{(\alpha_{0,I}^{(L)} + \alpha_{0,I}^{(NL)}) + \frac{k^3}{6\pi\epsilon_o}\left[(\alpha_{0,r}^{(L)} + \alpha_{0,r}^{(NL)})^2 + (\alpha_{0,I}^{(L)} + \alpha_{oI}^{(NL)})^2\right]}{\left[1 + \frac{k^3}{6\pi\epsilon_o}(\alpha_{0,I}^{(L)} + \alpha_{0,I}^{(NL)})\right]^2 + \left[\frac{k^3}{6\pi\epsilon_o}(\alpha_{0,r}^{(L)} + \alpha_{0,r}^{(NL)})\right]^2} \tag{S15}$$

By inserting equations S7,S8,S10, and S11 into S14 and S15, we can get the final equations for effective polarizability.

Our formulation can be applied to any type of particle including, non-absorbing, weakly absorbing, and highly absorbing particles. For non-absorbing particles ($\chi_1'' = 0, \chi_3'' = 0$), they can be simplified as follows:

$$\alpha_1' = 4\pi\epsilon_o R^3 \frac{\epsilon_p' - 1}{\epsilon_p' + 2} + 36\pi\epsilon_o R^3 E^2 \chi_3' \frac{\epsilon_p' - 1}{(\epsilon_p' + 2)^2} \tag{S16}$$

$$\alpha_1'' = \frac{8}{3}\pi\epsilon_o k^3 R^6 (\frac{\epsilon_p' - 1}{\epsilon_p' + 2})^2 + 48\pi\epsilon_o k^3 R^6 E^2 \chi_3' \frac{\epsilon_p' - 1}{(\epsilon_p' + 2)^3} \tag{S17}$$

In this case, Kerr nonlinearity (χ_3') appears in both real parts and imaginary parts of effective polarizability. It means it influences both the gradient and scattering forces involved in optical trapping. However, its impact on the gradient force is more pronounced leading to increasing trapping stability. This result shows that Kerr nonlinearity improves the stability of nonabsorbing nanoparticles. This observation is consistent with the results reported in [2].

In low absorption limit where ($\chi'' \ll \chi'$, $\chi_3'' \ll \chi_3'$), we get

$$\alpha' = \alpha_1' \tag{S18}$$

$$\alpha'' = \alpha_1'' + 12\pi\epsilon_0 R^3 \frac{\chi'' + 3E^2\chi_3''}{(\epsilon_p' + 2)^2} \tag{S19}$$

In this condition, the Kerr nonlinearity contributes to both gradient and scattering forces. Conversely, TPA solely contributes to the scattering force. The ratio of the Kerr effect to TPA components in equation S19 is proportional to $(kR)^3 \left(\frac{\chi_3'}{\chi_3''}\right)$, where $(kR)^3 \ll 1$ and $\left(\frac{\chi_3'}{\chi_3''}\right) \gg 1$. As a result, in certain circumstances, the Kerr effect dominates, whereas in others, TPA primarily contributes to the scattering force. Depending on the values of χ_3' and χ_3'', as well as the magnitude of the input power, the Kerr effect and TPA effects can either increase or decrease the gradient and scattering forces. Hence, the new potential traps other than the harmonic quadratic potential can be achieved.

From equations S18 to S19, both the real and imaginary components of effective polarizability can be formulated by a combination of linear and nonlinear components. Therefore, the gradient and scattering forces can also be depicted as the summation of their linear and nonlinear counterparts. This suggests that we can decompose the system into two coupled

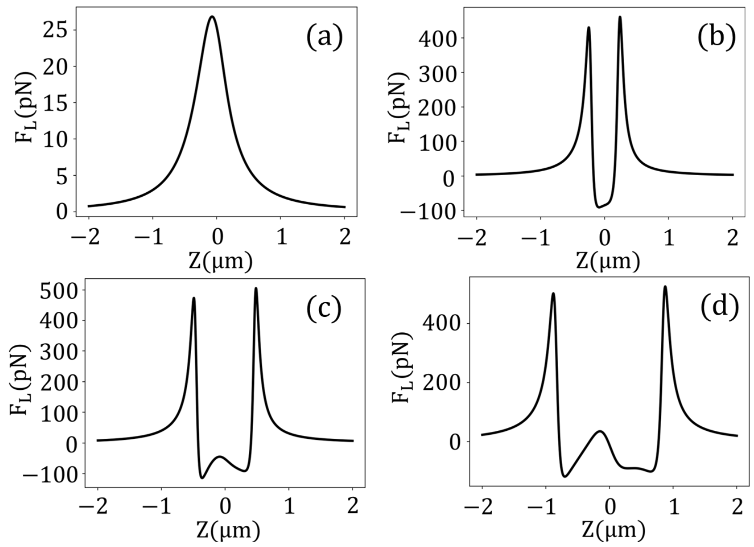

FIG. S1: The longitudinal force for (a) $P_{ave} = 100mW$, (b) $P_{ave} = 500mW$ (c) $P_{ave} = 1000mW$, and (d) $P_{ave} = 2500mW$, when considering both four-wave mixing and two-photon absorption

linear and nonlinear oscillators. Later, we provide a physical interpretation of the nonlinear oscillator by defining its parameters in terms of linear and nonlinear susceptibility.

Figure S1 shows the longitudinal forces at different powers when considering both the Kerr effect and two-photon absorption. At lower average powers (linear regime), the longitudinal force is positive on both the left and right sides of the focal point, i.e. the trap is unstable. As the power increases, two turning points with high slopes appear; the left zero-force point is the stable point with positive stiffness, and the second point is the unstable point with negative stiffness. Further increasing the power causes the distance between these zero-force points to increase. At extremely high powers, another zero-point force with positive stiffness tends to appear in the center which leads to a potential depth in the center. However, the potential depth at this point is very shallow.

Figure S2 shows the transverse forces at varying powers when accounting for both the Kerr effect and two-photon absorption. At low average powers (Figure S2(a)), the transverse force resembles the linear force with a positive stiffness representing a stable point at the focal point. As the power increases (Figure S2(b)), two zero-force points emerge on the left and right sides of the center, corresponding to the two split trapping wells (nonlinear traps). At higher powers (Figure S2(c)), three zero-force points become apparent: one is in the center (linear trap), and the other two are located on the left and right sides of the center,

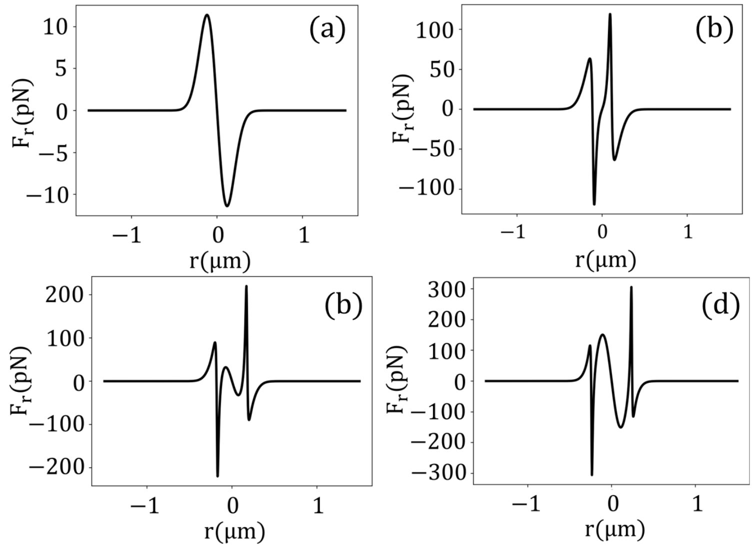

FIG. S2: The transverse force for (a) $P_{ave} = 100mW$, (b) $P_{ave} = 500mW$ (c) $P_{ave} = 1000mW$, and (d) $P_{ave} = 2500mW$, when considering both four-wave mixing and two-photon absorption.

respectively. At extremely high powers, the stiffness of the central trap is enhanced, while the stiffness of the side traps remains unchanged. Moreover, the slopes around the nonlinear trap points are much larger than their counterpart at the central point, which indicates that the stiffness of nonlinear traps is much stronger than that of the central linear trap.

Figure S3 shows the longitudinal forces acting on gold nanoparticles in air (inserts a and b) and water (insets c and d) when considering only a Kerr nonlinearity. As the power increases, the longitudinal force splits into two branches; however, in both mediums, no zero-force point (equilibrium point) occurs along the axial direction. Therefore, the Kerr effect does not lead to longitudinal trap stability.

Figure S4 shows the transverse forces acting on gold nanoparticles in air (inserts a and b) and water (insets c and d) at high powers when considering only a Kerr nonlinearity. As shown, in air the transverse force resembles the linear force. Although, the transverse force is perturbed at high powers, however, the linear shape is preserved. On the other hand, in water, at $P = 500mW$, two zero-point forces appear on the left and right sides of the focal point which correspond to two shallow potential depths. At high powers, three zero-force points become evident: one is at the center, and the other two are located on the left and right sides of the center. These correspond to three-split traps. These trap points

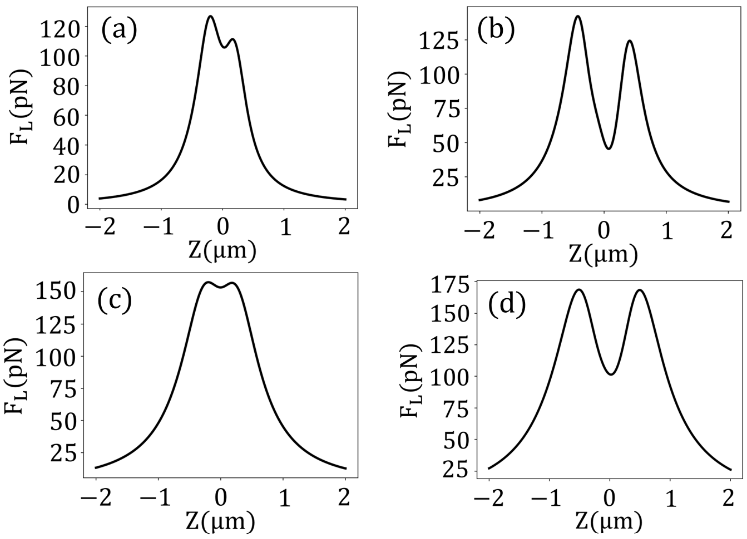

FIG. S3: The longitudinal force for (a) $P_{ave} = 500mW$, (b) $P_{ave} = 1000mW$ in air and (c) $P_{ave} = 500mW$, and (d) $P_{ave} = 1000mW$ in water, when considering only Kerr effect.

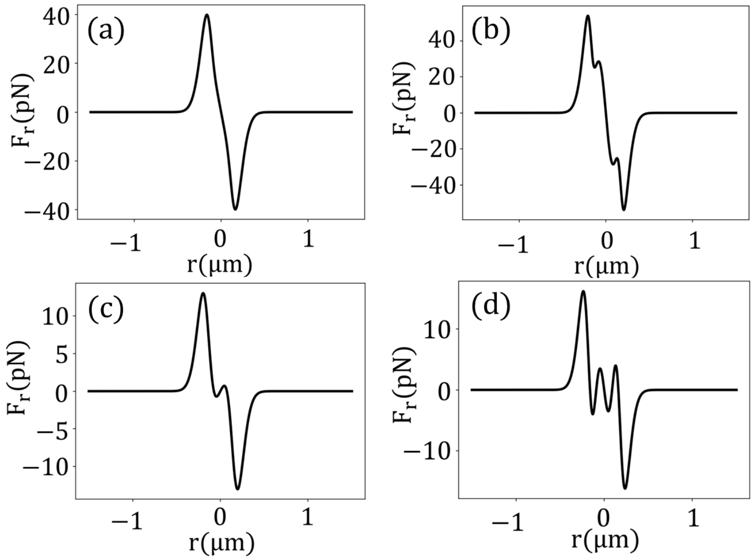

FIG. S4: The transverse force for (a) $P_{ave} = 500mW$, (b) $P_{ave} = 1000mW$ in air and (c) $P_{ave} = 500mW$, and (d) $P_{ave} = 1000mW$ in water, when considering only Kerr effect.

are shallow and are not sufficient to achieve a stable trap.

II. PHYSICAL INTERPRETATION

In this section, we provide a physical interpretation of the nonlinear trapping system. In the transverse plane, we model the nonlinear system modeled by the bistable potential and

illustrate the linear and nonlinear stiffnesses in terms of the linear and nonlinear susceptibilities. A similar approach can be used to model the potential trap with a tri-stable potential well. The nonlinear potential at distances between off-center points can be expressed by $U_{bi} = -\frac{1}{2}k_1 x^2 + \frac{1}{4}k_2 x^4$, where k_1 and k_2 respectively are the linear and nonlinear stiffnesses. The minimum points of this potential ($x = \pm\sqrt{\frac{k_1}{k_2}}$) are equivalent to the off-center trap points. To have a better insight into the mechanism of nonlinear optical trapping, we make some further simplifications. Here, we ignore the scattering force and self-induced back-action. These are reasonable assumptions for weakly absorbing particles experiencing a smaller scattering force in the transverse plane. In this case, the gradient potential reads $U = -\Re(\frac{\chi}{\chi+3})|\vec{E}|^2$ where $\vec{E} = \vec{E_0}e^{-\rho^2}$ is the Gaussian electric field with maximum amplitude of E_0, and total susceptibility is $\chi = \chi_1 + 3\chi_3 E_0^2 e^{-2\rho^2}$. Moreover, the real and imaginary parts of total susceptibilities can be written as $\chi' = \chi_1' + \Delta\epsilon_3' e^{-2\rho^2}$ and $\chi'' = \chi_1'' + \Delta\epsilon_3'' e^{-2\rho^2}$ in which $\Delta\epsilon_3' = 3\chi_3' E_0^2$ and $\Delta\epsilon_3'' = 3\chi_3'' E_0^2$.

After simplifications, the potential can be expressed by:

$$U = -E_0^2 e^{-2\rho^2} + \frac{3(3+\chi')}{(3+\chi')^2 + (\chi'')^2} E_0^2 e^{-2\rho^2} \tag{S20}$$

The total potential is expressed by the combination of linear (first term) and nonlinear (second term) potentials, i.e. $U = U_L + U_{NL}$. The linear part has a minimum point at $\rho = 0$ where its depth is $U_L(0) = -E_0^2$. On the other hand, the nonlinear potential reads

$$U_{NL} = \frac{3(3+\chi_1' + \Delta\epsilon_3' e^{-2\rho^2})}{(3+\chi_1' + \Delta\epsilon_3' e^{-2\rho^2})^2 + (\chi_1'' + \Delta\epsilon_3'' e^{-2\rho^2})^2} E_0^2 e^{-2\rho^2} \tag{S21}$$

Its maximum happens when $e^{-2\rho^2}$ is eliminated which means it happens at $\rho = 0$ with the maximum potential of

$$U_{NL}(0) = \frac{3(3+\chi_1' + 3\chi_3' E_0^2)}{(3+\chi_1' + 3\chi_3' E_0^2)^2 + (\chi_1'' + 3\chi_3'' E_0^2)^2} E_0^2. \tag{S22}$$

On the other hand, the minimum happens when $3 + \chi_1' + 3\chi_3' e^{-2\rho^2} = \pm(\chi_1'' + 3\chi_3'' e^{-2\rho^2})$. When considering $3 + \chi_1' + 3\chi_3' e^{-2\rho^2} = (\chi_1'' + 3\chi_3'' e^{-2\rho^2})$, the extremum points happen at $\rho^2 = \ln(\frac{3(\chi_3' - \chi_3'')E_0^2}{\chi_1'' - \chi_1' - 3})^{\frac{1}{2}}$ and when considering $3 + \chi_1' + 3\chi_3' e^{-2\rho^2} = -(\chi_1'' + 3\chi_3'' e^{-2\rho^2})$, the extremum points happen at $\rho^2 = \ln(\frac{\chi_1'' + \chi_1' + 3}{3(\chi_3' + \chi_3'')E_0^2})^{\frac{1}{2}}$. When the arguments of the logarithmic functions are larger than 1, we have the correct solutions and the off-center wells can appear. Therefore, whether split traps happen or not depends on the values of the linear and

nonlinear susceptibilities and the input power. In our case, for ($\chi_3' > 0$,$\chi_3'' < 0$, $\chi_1' < 0$, and $\chi_1'' > 0$), the first extremum point is correct. Thus, the off-center trap points happen at

$$\rho_{\pm} = \pm\sqrt{\ln(\frac{3(\chi_3' - \chi_3'')E_0^2}{\chi_1'' - \chi_1' - 3})^{\frac{1}{2}}} \tag{S23}$$

And the condition for happening split traps is: $\frac{3(\chi_3' - \chi_3'')E_0^2}{\chi_1'' - \chi_1' - 3} > 1$. Finally, the depth of nonlinear potential at off-center points is equal to

$$U_{NL}(\rho_{\pm}) = \frac{1}{2}\frac{\chi_1'' - (\chi_1' + 3)}{\chi_1''\chi_3' - \chi_3''(3 + \chi_1')} \tag{S24}$$

Then, by comparing the bistable potential (U_{bi}) with the trap potential (U) we can find the relation between linear and nonlinear stiffnesses as follows:

$$\frac{k_1}{k_2} = \ln(\frac{3(\chi_3' - \chi_3'')E_0^2}{\chi_1'' - \chi_1' - 3})^{\frac{1}{2}} \tag{S25}$$

[1] L. Novotny and B. Hecht, *Principles of nano-optics* (Cambridge university press, 2012).

[2] A. Devi and A. K. De, "Generalized description of the nonlinear optical force in laser trapping of dielectric nanoparticles," Physical Review Research **2**, 043378 (2020).

[3] S. Mirzaei-Ghormish, S. Duke, and D. E. Smalley, "Nonlinear Optical Trap Modeling of Rayleigh Particles," in *3D Image Acquisition and Display: Technology, Perception and Applications* (Optica Publishing Group, 2023) pp. DW5A–7.
